# P-700. Examining the Knowledge, Availability, Access and Uptake of RSV Immunoprophylasis among RSV Hospitalized Infants

**DOI:** 10.1093/ofid/ofae631.896

**Published:** 2025-01-29

**Authors:** Jamee T Shelley, Tyler Walsh, Lisa Gorham, Erika Madrian, Hannah Neiditz, Ana V Torres, Jasmine Prater, Megan D Heroux, David Catherall, Brian Sun, Humza Agha, meghan Ott, Kelly True, Lori B Barganier, Terrill M Cindy, Patrick J Reich, Jason G Newland

**Affiliations:** Washington University School of Medicine, Canal Winchester, Ohio; Washington University School of Medicine in Saint Louis, St. Louis, Missouri; Washington University School of Medicine, Canal Winchester, Ohio; Washington University School of Medicine, Canal Winchester, Ohio; Washington University School of Medicine in St Louis, St Louis, Missouri; Washington University in St. Louis School of Medicine, St. Louis, Missouri; Washington University School of Medicine, Canal Winchester, Ohio; Washington University in St.Louis School of Medicine, Saint Louis, Missouri; Washington University in St. Louis, Saint Louis, Missouri; Washington University School of Medicine, Canal Winchester, Ohio; Washington University in St. Louis School of Medicine, St. Louis, Missouri; Washington University in St Louis School of Medicine, St. Louis, Missouri; Washington University In ST. Louis, St. Louis, Missouri; Washington University St. Louis, St Louis, Missouri; Washington University in St. Louis, Saint Louis, Missouri; Washington University School of Medicine, Canal Winchester, Ohio; Washington University in St. Louis School of Medicine, St. Louis, Missouri

## Abstract

**Background:**

Every year, up to 80,000 children less than 5 years are hospitalized due to RSV infection and those less than 1 are at greatest risk for ICU admission. In the Fall of 2023, 2 immunoprophylaxis strategies, nirsevimab for infants and Abrysvo for pregnant women, were approved to prevent serious RSV infection in infants. This study investigates whether caregivers of infants admitted with RSV were aware, had access of RSV and whether the RSV vaccine was received.

**Table 1:**
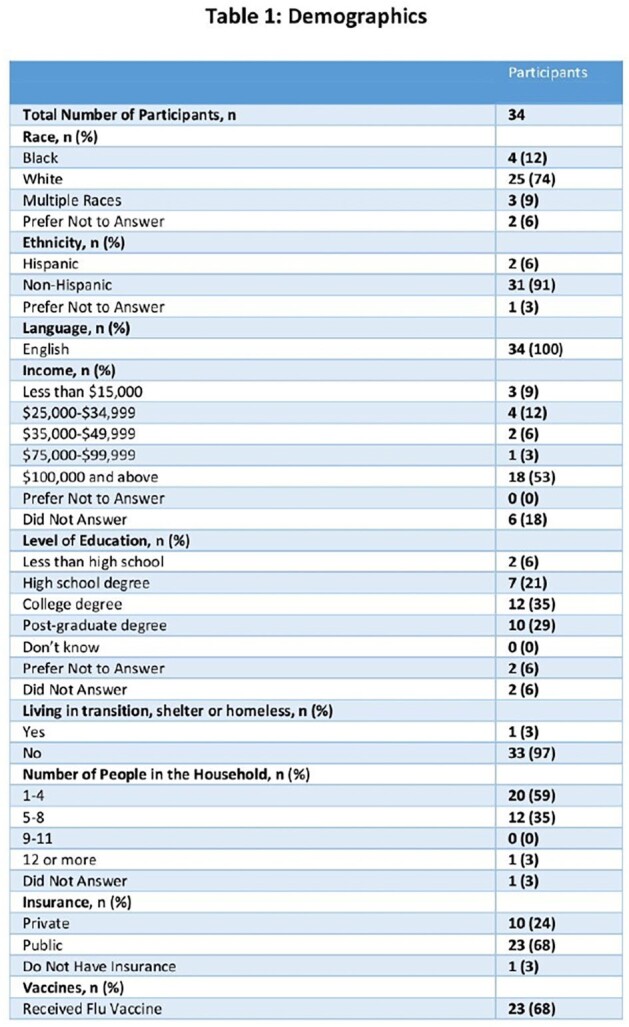
RSV Respondents Demographics

**Methods:**

A survey study was conducted among families with infants born between April 2023 and March 2024, who were admitted to St. Louis Children's Hospital and tested positive for RSV. The survey asked questions among caregivers regarding the main themes: awareness, availability and uptake of the immunoprophylaxis.

**Table 2:**
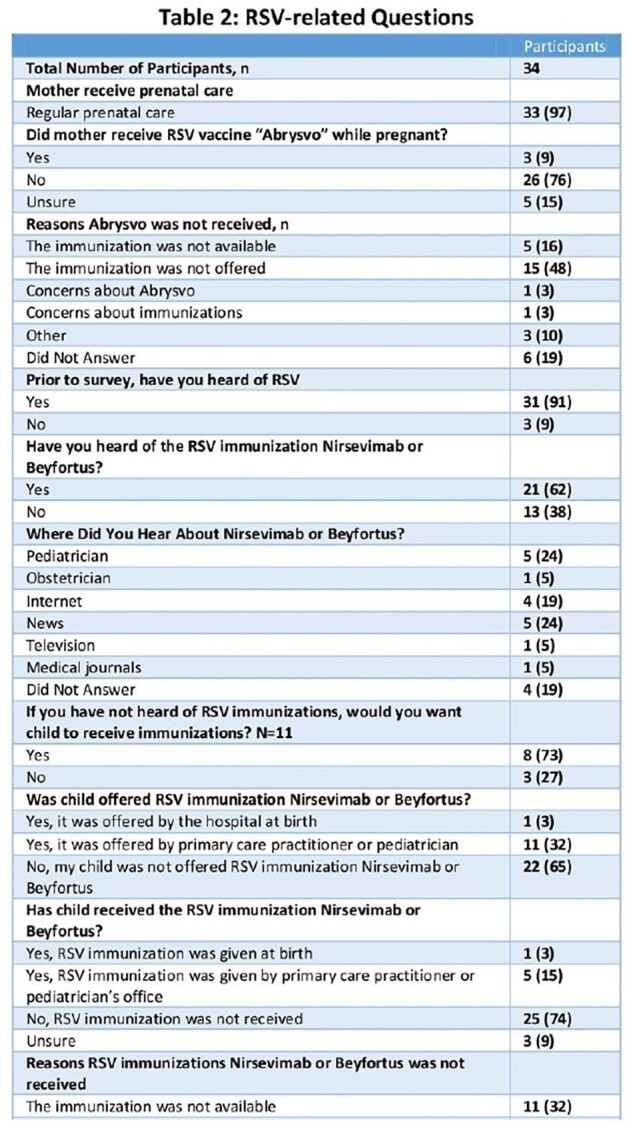
RSV-related Caregiver Responses

**Results:**

A total of 34 surveys were completed with 3 moms having received Abrysvo and 6 infants having received nirsevimab. The demographics of the respondents were 12% Black, 74% White, and 9% Multiple races; and 6% Hispanic (Table 1). Of those receiving immunoprophylaxis, 5 and 4 had public and private insurance, respectively (Figure 1). Of the 97% of mothers that received regular prenatal care, only 9% received Abrysvo (Table 2). Of the 31 mom not receiving Abrysvo, 16% responded that the RSV vaccine was not available and 48% responded that the vaccine was not offered (Table 2). Of those infants with RSV who had not received nirsevimab, 65% were not offered the immunization, nirsevimab and Beyfortus. Of those not offered nirsevimab, 32% reported it was not available, 18% did not know about it, and 2% of moms received Abrysvo (Table 2).

**Figure 1:**
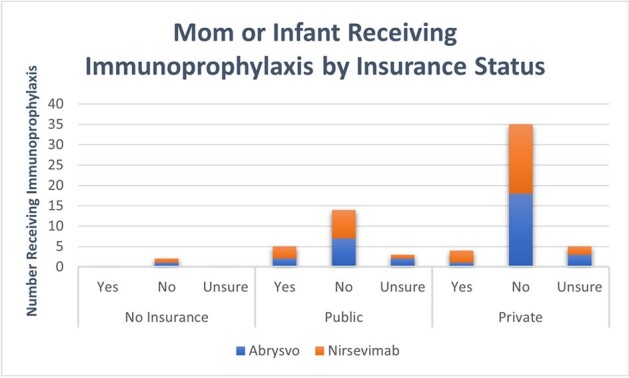
Mom or Infant Receiving Immunoprophylaxis by Insurance Status

**Conclusion:**

For those moms and infants not receiving Abrysvo or nirsevimab, awareness and availability were important reason. In the upcoming RSV season, more efforts are needed to ensure these factors are addressed in delivering these important immunoprophylaxis products.

**Disclosures:**

**Jason G. Newland, MD, MEd**, Moderna: Grant/Research Support|Pfizer: Grant/Research Support

